# The synergistic effects of soil-applied boron and foliar-applied silicon on cotton fiber quality and yield

**DOI:** 10.1186/s12870-023-04542-y

**Published:** 2023-10-27

**Authors:** Priscilla Maria da Silva Liber Lopes, Cid Naudi Silva Campos, Paulo Eduardo Teodoro, Milton Garcia Costa, Jonas Pereira de Souza Júnior, Renato de Mello Prado

**Affiliations:** 1 Federal University of Mato Grosso Do Sul (UFMS), Rod MS 306, Km 105 S/N, Chapadão Do Sul, Brazil; 2 Department of Agricultural Production, São Paulo State University ‘Júlio de Mesquita Filho’ (UNESP), School of Agricultural and Veterinarian Sciences, Via de Acesso Prof. Paulo Donato Castellane S/N, Jaboticabal, 14884900 Brazil; 3 Federal Institute of São Paulo (IFSP). Rod Nadir Kenan, Km 03 S/N, Barretos, 14781-502 Brazil

**Keywords:** *Gossypium hirsutum*, Micronutrient, Beneficial element, Micronaire

## Abstract

Studies of boron (B) and silicon (Si) synergy in cotton crops have shown promising results; however, the focus was on the foliar application of B and Si. Nonetheless, B is an element with little mobility in the plant and its best form of application is in the soil. Thus, the objective of this study was to evaluate the synergistic effect of soil applied B and foliar applied sSi on fiber quality and crop yield of cotton. For this purpose, a field experiment was carried out using cotton cultivar FM 985 GLTP. The soil’s B in the experimental site is classified as low for cotton cultivation. The experiment was conducted in a randomized complete-block design, in a 3 × 2 factorial scheme, with three doses of B: 0.0 kg ha^−1^ (deficiency), 2.0 kg ha^−1^ (recommended dose), and 4.0 kg ha^−1^ (high dose) in the absence and presence (920 g L^−1^) of Si, with four replications. One week after the 4^th^ application of Si, B and Si leaf content was determined. At boll opening, crop yield was estimated, and fiber quality analysis was realized. Boron deficiency reduced cotton yield, in 11 and 9%, compared to the application of 2 and 4 kg ha^−1^ of B, respectively. The presence of Si, however, increased plant yield in 5% in the treatments with 0 and 2 kg ha^−1^ of B, respectively. Cotton fiber length and elongation were not influenced by the B doses and Si presence. Fiber breaking strength was increased in 5% by the presence of Si and was not influenced by B deficiency. Micronaire was 8% smaller in the treatment with 0 kg ha^−1^ of B and 6% smaller in the absence of Si. Short fiber index was 4% greater in the plants of the treatment with 0 kg ha^−1^ of B. The results of this study reports that the complementation with Si via foliar application increases fiber quality by enhance breaking strength and micronaire. In conclusion, the interaction between soil-applied B and foliar-applied Si is beneficial for cotton cultivation, resulting in high cotton yield with better fiber quality.

## Introduction

The availability of sufficient and balanced nutrition plays a significant role in influencing cotton yield and quality [1–4]. Deficiencies in macro- and micronutrients can negatively impact both vegetative and reproductive growth, leading to reduced fiber cotton yield [3, 5]. Boron (B) deficiency, in particular, can significantly affect cotton yield and fiber quality, since B-deficiency is a common problem in cotton-producing regions, mainly in tropical soils, where B availability is impaired due to low contents of organic matter and clay, reducing yield [6] and impairing cotton fiber quality [6, 7].

The application of B via soil is a common practice in cotton crop cultivation, improving cotton development, physiological parameters and fiber yield and quality [6, 8–11]. Additionally, B application via soil has been recommended for higher yield and productivity in cotton crop [12]. In B-deficient soils, the application of silicon (Si), via foliar, emerges as an innovative strategy, which may have a synergistic effect with B, because these elements have similarities and are both absorbed in molecular form as boric and monosilicic acids, respectively [13, 14]. Preliminary studies, under controlled conditions, indicate that Si may attenuate B deficiency by acting in the production of non-enzymatic antioxidant compounds, reducing oxidative stress, and increasing plant biomass production [15–17]. Under field conditions, recent studies, provide evidence that the application of this beneficial element can improve cotton development [18, 19], and also assists in increasing cotton fiber yield and quality, with higher micronaire, fiber length, tensile strength, and a lower percentage of short fibers [7].

Studies about B and Si synergy in cotton crops have shown promising results; however, the focus was on the foliar application of these elements [7, 15, 16]. Nonetheless, B is an element with little mobility in the plant and its best form of application is in the soil, expanding the crop response [20]. Some studies have demonstrated the significance of maximizing the synergy of these elements by supplementing Si via foliar treatment with B via the soil to boost plant uptake [21, 22]. Therefore, further studies are important to optimize the synergy of these elements using B via the soil to increase plant absorption, complementing with Si via foliar application, in cotton crops cultivated under tropical condition.

Thus, our hypothesis is that the Si via foliar application can boost the soil applied B, and significantly effected in cultivation of cotton crop under tropical conditions. It is expected that foliar-Si in the reproductive phase of the cotton plant can have a summation effect, increasing crop yield and, mainly, fiber quality.

This study investigated the increas in crop yield and fiber quality of cotton cultivated with soil-applied B, associated to foliar-applied Si. In view of the advancement of cotton cultivation in tropical regions in B-deficient soils and the need to improve fiber quality, field studies are crucial to prove the synergy between B and Si, which enables sustainable cotton cultivation to reconcile yield and quality in a balanced way, a major challenge for future agriculture.

## Materials and methods

The field experiment was carried out during October, 2019—March, 2020 at Fundação Chapadão in the municipality of Chapadão do Sul, Mato Grosso do Sul State, Brazil (18°41′33″ S and 52°40′45″ W), using cotton cultivar FM 985 GLTP.

**Fig. 1:**
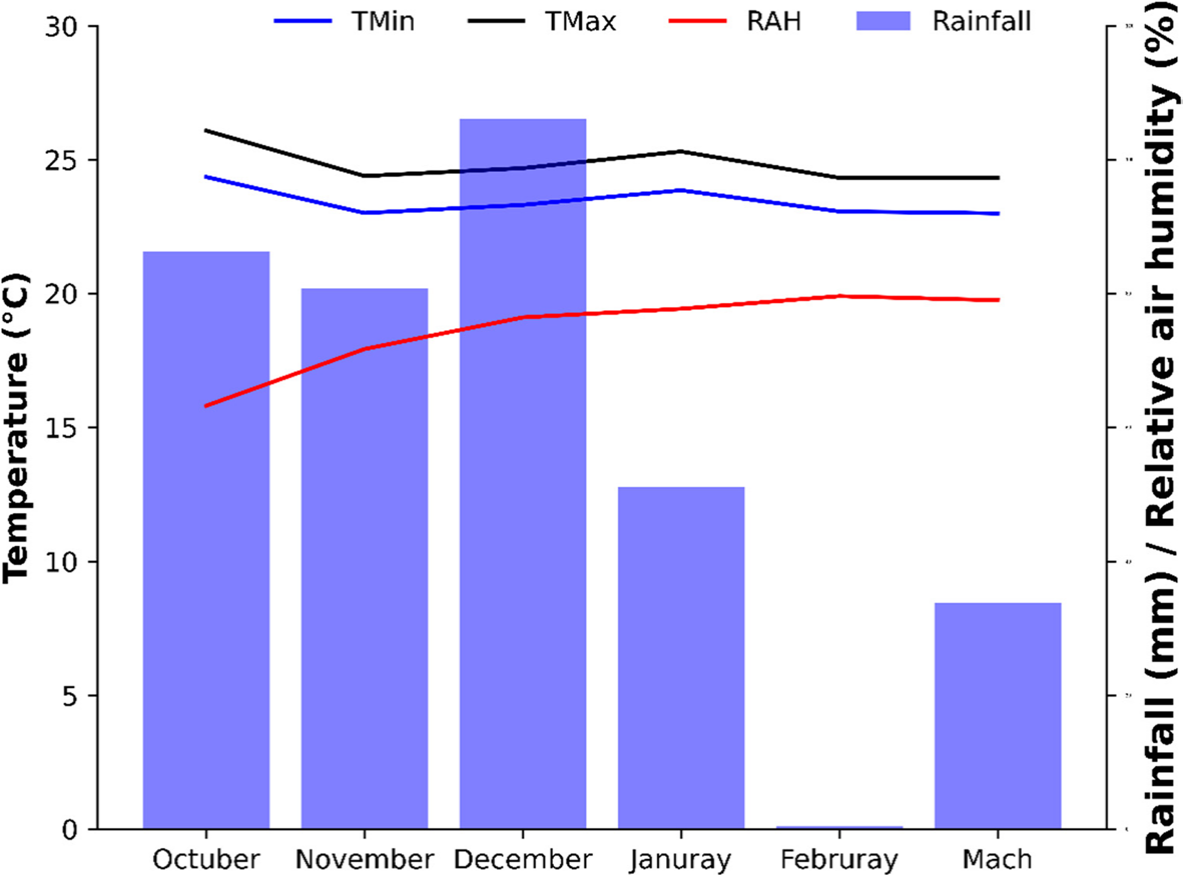
Maximum (TMax) and minimum (Tmin) temperature, relative air humidity (RAH) and rainfall at Fundação Chapadão during the experimental period

The climate of the region is classified as humid tropical type (Aw), with a rainy season in the summer and a dry season in the winter, and an average annual rainfall of 1850 mm. The annual temperature ranges from 13 to 28 °C (daily measurements). Maximum and minimum temperature, relative air humidity and rainfall during the experimental period were recorded using a thermohydrometer (Fig. 1).

The total precipitation obtained during the experiment was 983.2 mm, meeting the range of water requirements for the cotton crop, which is between 500 a 1500 mm of annual rainfall and a relative humidity of around 60%. Regarding thermal requirements, both minimum and maximum temperatures were within the ideal range for the crop, around 15 to 30 °C [23]

The soil in the experimental site was classified as Dystrophic Red Latosol [24]. Before the experiment was set up, soil sampling was conducted at a depth of 0.00 to 0.20 m, followed by soil chemical analysis for fertility purposes [25], presenting the following results: pH (CaCl_2_): 4.9; P(Res): 74.60 mg dm^−3^; organic matter: 31.80 g dm^−3^; K: 133.00 mg dm^−3^; Ca: 3.20 cmol_c_ dm^−3^; Mg: 0.80 cmol_c_ dm^−3^; S: 25.50 mg dm^3^; H + Al: 5.30 cmol_c_ dm^−3^; Al: 0.10 cmol_c_; B: 0.30 mg dm^−3^; Cu: 1.20 mg dm^−3^; Fe: 47 mg dm^−3^; Mn: 17.30 mg dm^−3^ e Zn: 5.60 mg dm^−3^; CEC: 9.64 cmol_c_ dm^−3^; V (%): 45. The particle size composition was also analyzed [26]: 63.0% clay, 29.5% sand, and 7.5% silt. It is noteworthy that soil’s B content is classified as low for cotton cultivation [12].

The area was prepared with soil correction, applying 1,500 kg ha^−1^ of limestone and 700 kg ha^−1^ of agricultural gypsum, broadcast without incorporation. After the limestone had a chance to react, a pre-seeding fertilization was carried out with 200 kg ha^−1^ of KCl (60% of K) broadcast. The experiment was sown on October 10, 2019, and in-row fertilization was performed with 270 kg ha^−1^ of a formulated fertilizer with 11% of N, 44% of P_2_O_5_ and 3% of S, as topdressing. At 25 days after emergence (DAE), it was done 200 kg ha^−1^ of urea (45% N) broadcast, and at 50 DAE with 350 kg ha^−1^ of ammonium sulfate (20% N and 24%S) broadcast.

The experiment was conducted in a randomized complete-block design, in a 3 × 2 factorial scheme, with three doses of B: 0.0 kg ha^−1^ (deficiency), 2.0 kg ha^−1^ (recommended dose - [12]), and 4.0 kg ha^−1^ (high dose) in the absence and presence (920 g ha^−1^) of Si, with four replications. The B source used was ulexite (100 g kg^−1^ of B and solubility in water was 950 g L^−1^ at 25 °C). The Si source was potassium silicate stabilized with sorbitol (SiKE—115 g L^−1^ of Si, 113.85 g L^−1^ of K_2_O, 100 mL L^−1^ of sorbitol and pH 12.0), while K was balanced between the different treatments.

The experimental plots consisted of four lines with 11 m spaced at 90 cm, totaling 39.6 m^2^ as plot area. The useful area used for evaluations consisted of two central lines of 6 m long, totaling 10.8 m^2^.

Boron application via the soil was carried out according to the treatments in the total area of each plot, manually and only once, in pre-emergence, using agricultural gypsum as filler in the proportion of 500 kg ha^−1^. Silicon was applied via the leaves with four applications, according to the treatments, starting at the B1 reproductive stage (one flower bud on the first reproductive branch) with intervals of 7–11 days, varying with climatic conditions. The application was carried out with a Herbicat® CO_2_ pressure sprayer, adjusted for an application rate of 200 L ha^−1^ of spray solution, with a service pressure of two bars, under a bar with four nozzles, spaced at 90 cm. Meteorological conditions at the time of the application were: temperature < 26 °C, relative air humidity > 60%, and wind speed < 8 km h^−1^, favorable for foliar application [20].

Crop phytosanitary management was carried out in accordance with technical recommendations [7]. For weed control, 3 L ha^−1^ of Glyphosate (588 g L^−1^) and 500 mL ha^−1^ of Thidiazuron (120 g L^−1^) were used. The insecticides used were 1.8 L ha^−1^ of Abamectin (18 g L^−1^), 200 g ha^−1^ of Acetamiprid (200 g kg^−1^), 1.8 L ha^−1^ of Carbosulfan (700 g L^−1^), 1.5 L ha^−1^ of Spiromesifen (240 g L^−1^), 2.25 L ha^−1^ of Etiprole (200 g L^−1^), 1 L ha^−1^ of Fipronil (250 g L^−1^), 100 g ha^−1^ of Imidacloprid (700 g L^−1^), 4.5 L ha^−1^ of Malathion (1000 g L^−1^), 1.6 L ha^−1^ of Methomyl (215 g L^−1^), 150 mL ha^−1^ of Novaluron (100 g L^−1^), 300 mL ha^−1^ of Pyriproxyfen (100 g L^−1^), 500 g ha^−1^ of Thiodicarb (800 g kg^−1^), and 3 L ha^−1^ of the mixture of Beta-cyfluthrin (12.5 g L^−1^) + Imidacloprid (100 g L^−1^). Additionally, growth regulators were applied according to the technical recommendations for each product.

One week after the 4^th^ application of Si, samples were collected from 10 leaves per plot (5^th^ leaf completely expanded from the apex). The samples were decontaminated and oven dried (65 ± 5 °C) before milling. Then, the chemical analysis of the leaves was conducted to determine B content. This was done by dry digestion in a muffle furnace at 400 °C for three hours, followed by a colorimetric reaction with H-Azomethine and subsequent colorimetric reading using a spectrophotometer [27]. Silicon content was determined by alkaline digestion with H_2_O_2_ and NaOH, followed by a colorimetric reaction with ammonium molybdate, and it was determined by colorimetric reading using a spectrophotometer [28].

At boll opening, the useful area was harvested, and crop yield was estimated by weighing the cotton bolls within the useful area (10.8 m^2^) extrapolating the production to 1 ha. After weighing, the samples were submitted to the fiber quality analysis, using the High Volume Instrument™ (HVI) in an environment with controlled temperature and humidity to measure micronaire, length, rupture resistance, short fiber index (SHI), and leaf elongation [29].

The data were submitted to the analysis of variance by the F test (p < 0.05) and normality verification (Shapiro–Wilk W test). The means were compared by the Tukey test at a 5% probability level, using the AgroEstat® software [30]. The multivariate analysis of canonical variables was performed to verify the interrelationship between treatments and variables. The statistical analyses were performed with free software R using the packages “factoextra”, “gplots” and “pheatmap”.

## Results and discussion

**Fig. 2:**
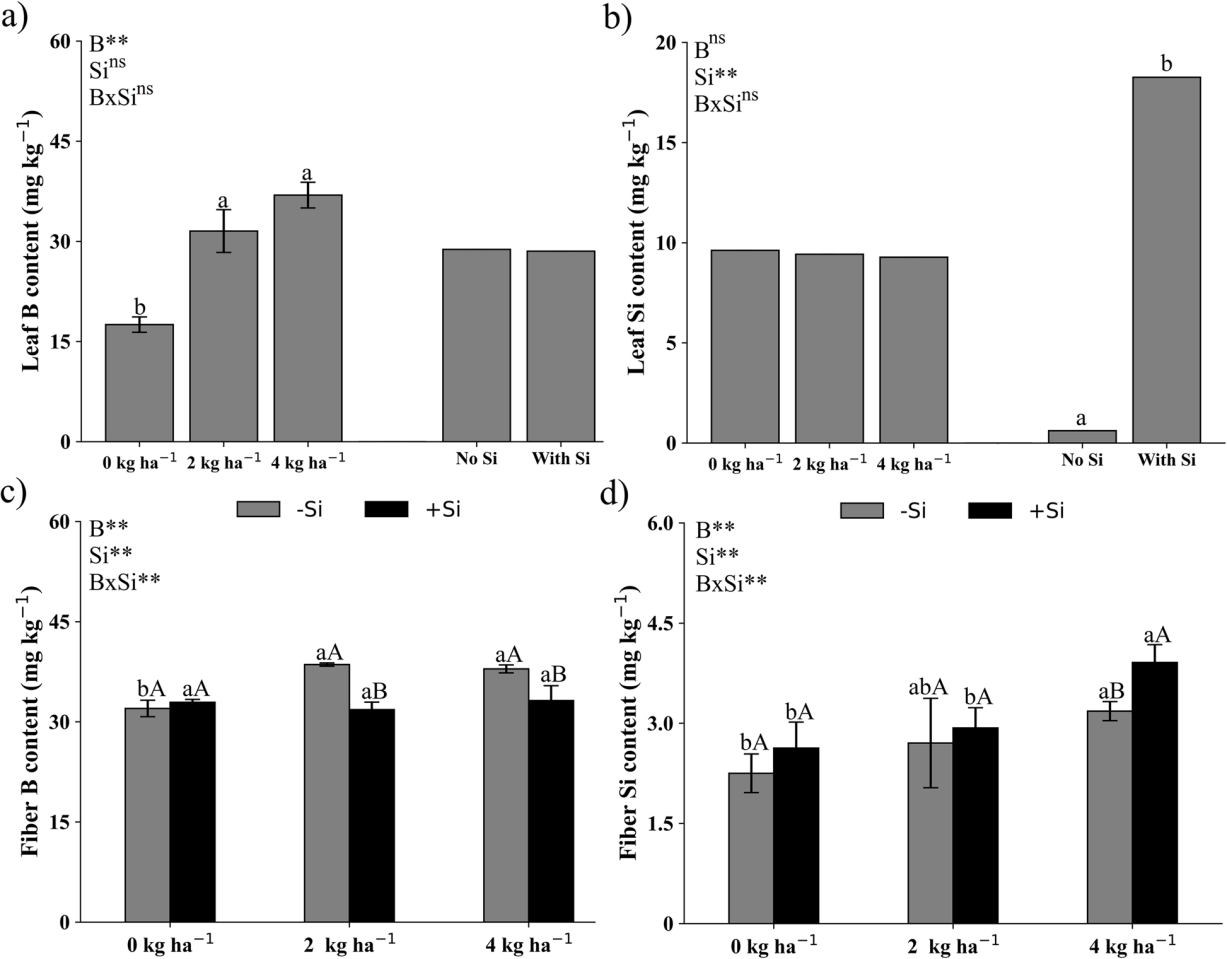
Leaf concentration of B (**a**) and Si (**b**) and cotton fiber concentration of B (**c**) and Si (**d**) cultivated under different doses of B applied via soil (D) in the presence and absence of silicon (Si) via foliar application. Small letters differ the B doses, while capital letters differ Si application by the Tukey test at 5% probability

In the leaves, there was no interaction between treatments for the contents of B (Fig. 2a) and Si (Fig. 2b). The increment in soil-applied B dose, to 2 and 4 kg ha^−1^, increased the B content in the leaf. The adequate content of foliar B in cotton plants is between 35 and 55 mg kg^−1^ of B [11], indicating that the plants in this study did not reach toxicity, even with the application of the double of the recommended dose for B (4 kg ha^−1^) (Fig. 2a).

The Si foliar application was efficient to increase leaf Si content in cotton plants (Fig. 2b). This result was expected, since the source and concentration used did not showed the Si polymerization process, and the use of sorbitol as stabilizer can significantly increase the efficiency of Si foliar application in cotton [7, 16, 31].

In cotton fibers, there was an interaction between treatments for the contents of B (Fig. 2c) and Si (Fig. 2d). The treatments with soil-applied B with Si applied via foliar, did not differ due to the increase in soil-applied B dose (Fig. 2c). On the other hand, in the treatments with soil-applied B with no Si applied via foliar, the treatment with 0 kg ha^−1^ of B resulted in the lowest micronutrient content compared to the other treatments with no Si applied via foliar. The Si foliar application did not increased the fiber B content in plants at 0 kg ha^−1^ B, conversely, at 2 and 4 kg ha^−1^ of B, Si foliar application reduced B concentration in the cotton fiber (Fig. 2c). For fiber Si content, the Si foliar application was effective only in the plants that received 4 kg ha^−1^ of soil applied B (Fig. 2d). Both B and Si are important for the formation and development of cotton fiber [4, 6, 7, 32]; thus, these results suggest that a substitution in the demand for B by Si in the cotton fiber may have occurred, but more specific studies need to be conducted.

**Fig. 3:**
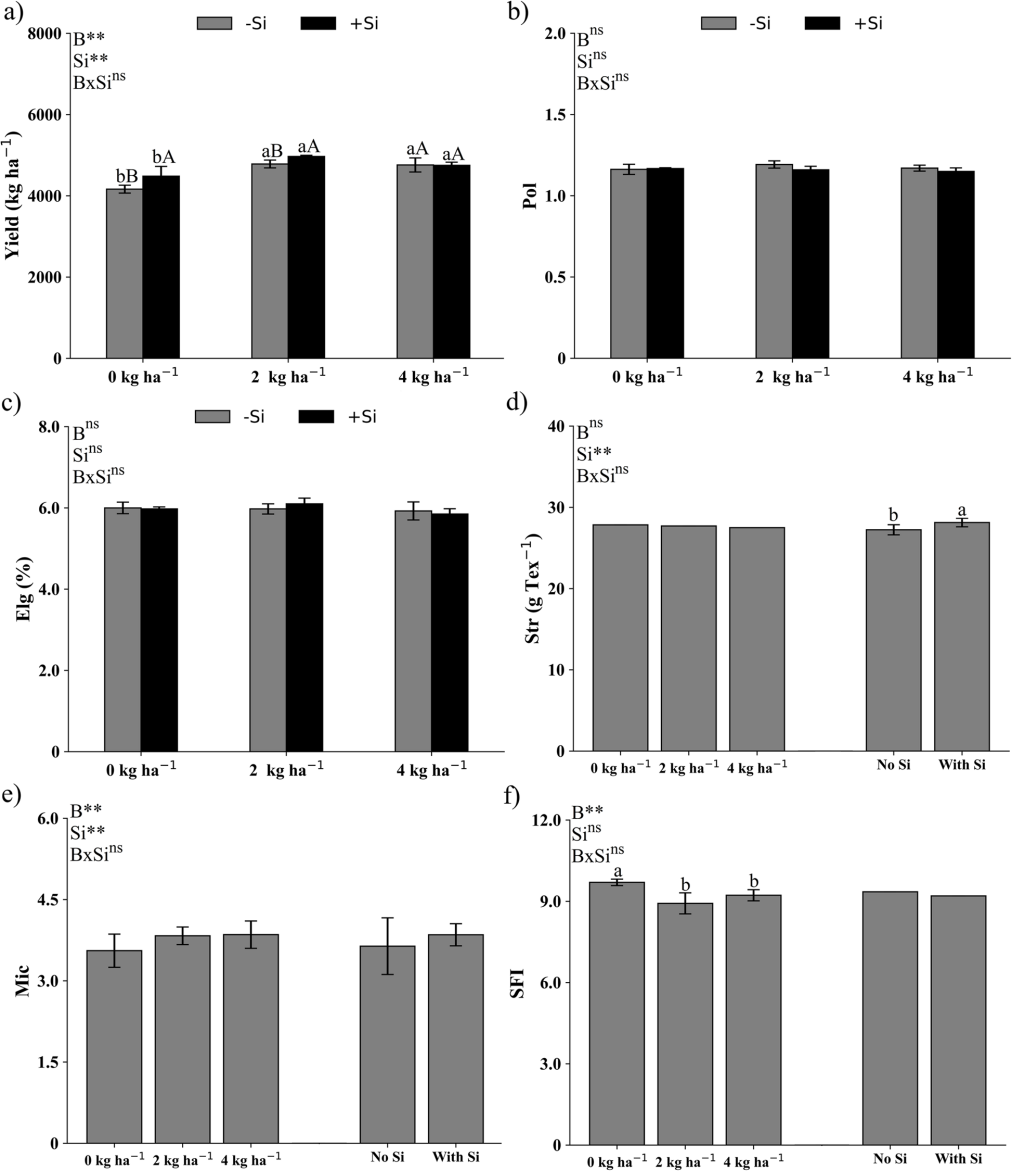
Yield (**a**), fiber length (Pol) (**b**), elongation capacity (Elg) (**c**), breaking strength (Str) (**d**), micronaire (Mic) (**e**, **f**) and short fiber index (SFI) (**g**) in cotton fiber cultivated under different doses of B applied via soil (D) in the presence and absence of silicon (Si) via foliar application. Small letters differ B doses, while capital letters differ application of Si by the Tukey test at 5% probability

Boron deficiency reduced cotton yield, while applications of 2 or 4 kg ha^−1^ of B via the soil did not show a difference (Fig. 3a). The presence of Si, however, increased plant yield only in the treatments with 0 and 2 kg ha^−1^ of B (Fig. 3a). Silicon has beneficial effects on plants, mainly under stressed conditions [33]. In cotton B-deficient plants Si has proven its efficiency in mitigating the deleterious effects of B deficiency in cotton plants by the reduction of oxidative stress and increasing the antioxidant compounds biosynthesis, resulting in greater plant growth and higher yields [7, 15, 16]. Nonetheless, Si also have proven that can increase development and productivity in cotton plants under no induced stress conditions by favoring photosynthetic parameters [18, 19].

The qualitative variables of the cotton fiber length (Pol – Fig. 3b) and elongation (Elg—Fig. 3c) were not influenced by the B doses and Si presence.

Fiber breaking strength (Str) was increased only by the presence of Si and was not influenced by B deficiency (Fig. 3d). Silicon plays an important structural role, forming complexes with calluses, tannins, and pectins in addition to establishing bonds with (1;3, 1;4)-β-D-glucan, which contributes to the strengthening of the cell wall [34], resulting in higher fiber breaking strength [7].

Micronaire showed an isolated effect of the treatments with no interaction between soil-applied B and Si foliar application. Micronaire was smaller in the treatment with 0 kg ha^−1^ of B and in the absence of Si (Fig. 3e). Micronaire, which is a measure of fiber fineness and maturity, is affected by B [4, 35] and Si [7, 36] nutrition. Boron can affect the flow of metabolites through the pyrimidine synthesis pathway [37], leading to micronaire formation. Silicon, is also important for micronaire development since it has several functions in cotton fiber development and maturity increasing micronaire index on cotton fiber [7, 38].

Short fiber index (SFI) presented an isolated effect of B and was greater in the plants of the treatment with 0 kg ha^−1^ of B (Fig. 3f). Boron plays a crucial role in the biosynthesis of essential components of cotton fibers such as cellulose, hemicellulose, pectin, and lignin [34], and B-deficiency negatively affects fiber compounds production and quality what can increasing the SFI [6].

**Fig. 4:**
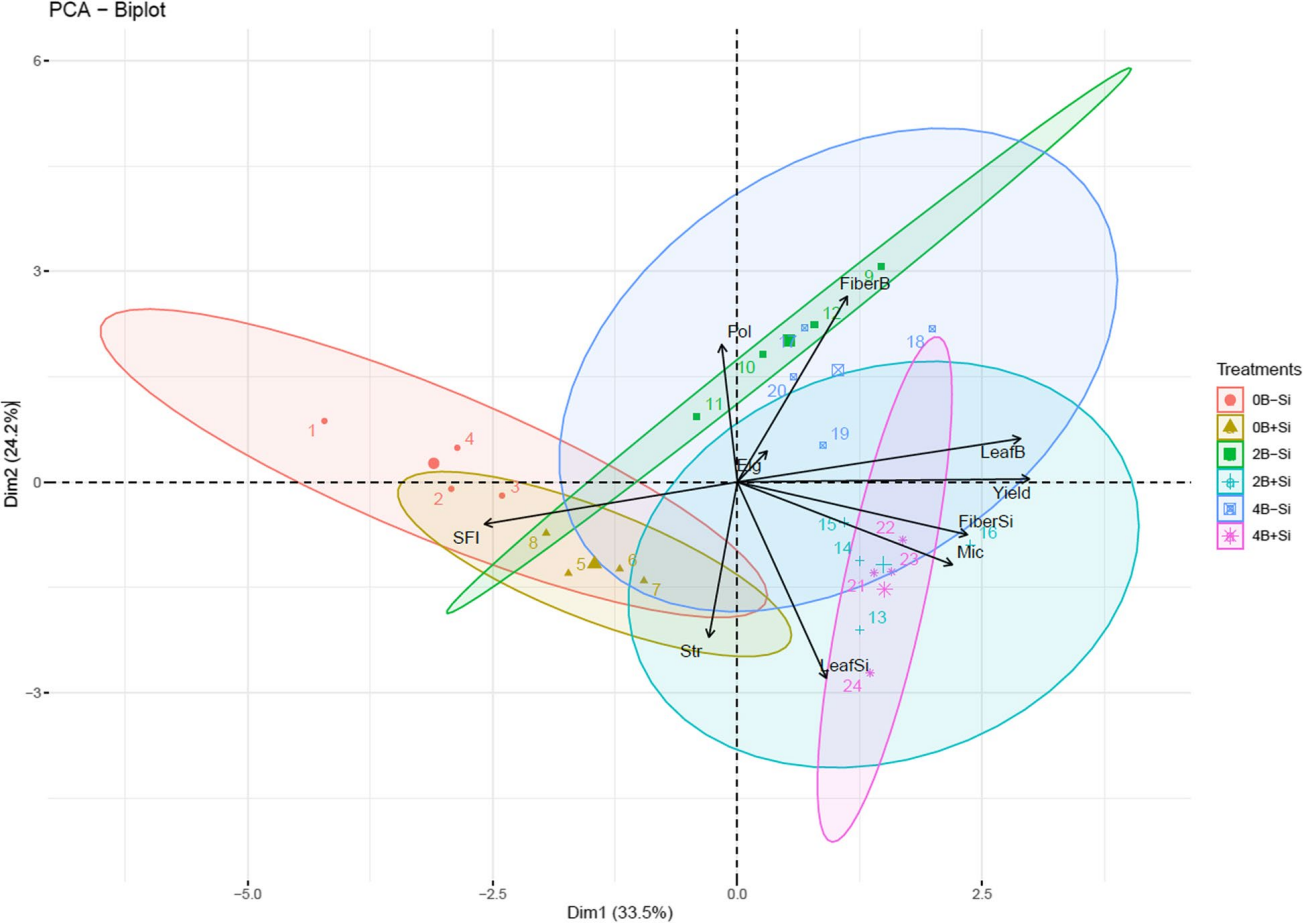
The principal component analysis (PCA) of yield, leaf B content, leaf Si content, B fiber content, Si fiber content, fiber length (Pol), fiber elongation capacity (Elg), breaking strength (Str), micronaire (Mic), and short fiber index (SFI) of cotton cultivated under different concentrations of B (0, 2, and 4 kg ha^−1^) applied via the soil in the presence (+ Si) and absence (-Si) of silicon (Si) applied via the leaves

The principal component analysis (PCA) indicated a greater association between SFI and treatments with 0 kg ha^−1^ of B in the absence and presence of Si (Fig. 4). As previously mentioned, B plays an important role in the synthesis of components that increase cotton fiber quality [7], and its deficiency is associated to an increase in SFI [6]. The treatment with 0 kg ha^−1^ of B also showed a greater association with Str in the presence of Si, highlighting the importance of Si to increase the fiber structural components, contributing to cell strengthening and, consequently, to greater Str.

Elg showed low contribution to explain the components analyzed by PCA and was consistent with the results observed in the univariate analysis, indicating that Elg is not negatively influenced by B deficiency or positively affected by Si foliar application.

Pol is a measure of fiber length, while Elg represents the fiber elongation capacity. The results of this study indicate that these variables are not negatively influenced by B deficiency or positively affected by Si foliar application.

Pol and the B content in the fiber showed a greater association with the treatment with 2 kg ha^−1^ of B in the absence and presence of Si (Fig. 3b).

The leaf Si content was associated to the treatment with 4 kg ha^−1^ in the presence of Si, while yield, Mic, and the B content in the leaf and Si in the fiber were more associated to the treatment with 4 kg ha^−1^ in the absence and presence of Si. The increase in B uptake, indicated by the B content in the leaf, and the greater presence of Si in the fiber increased yield and fiber quality of the cotton plant. This result indicates a summation effect in these treatments. These variables are inverse to the SFI, demonstrating that the increases of the leaf content of B and the Si content in the cotton fiber are related to the reduction of this derogatory variable of the fiber.

**Fig. 5:**
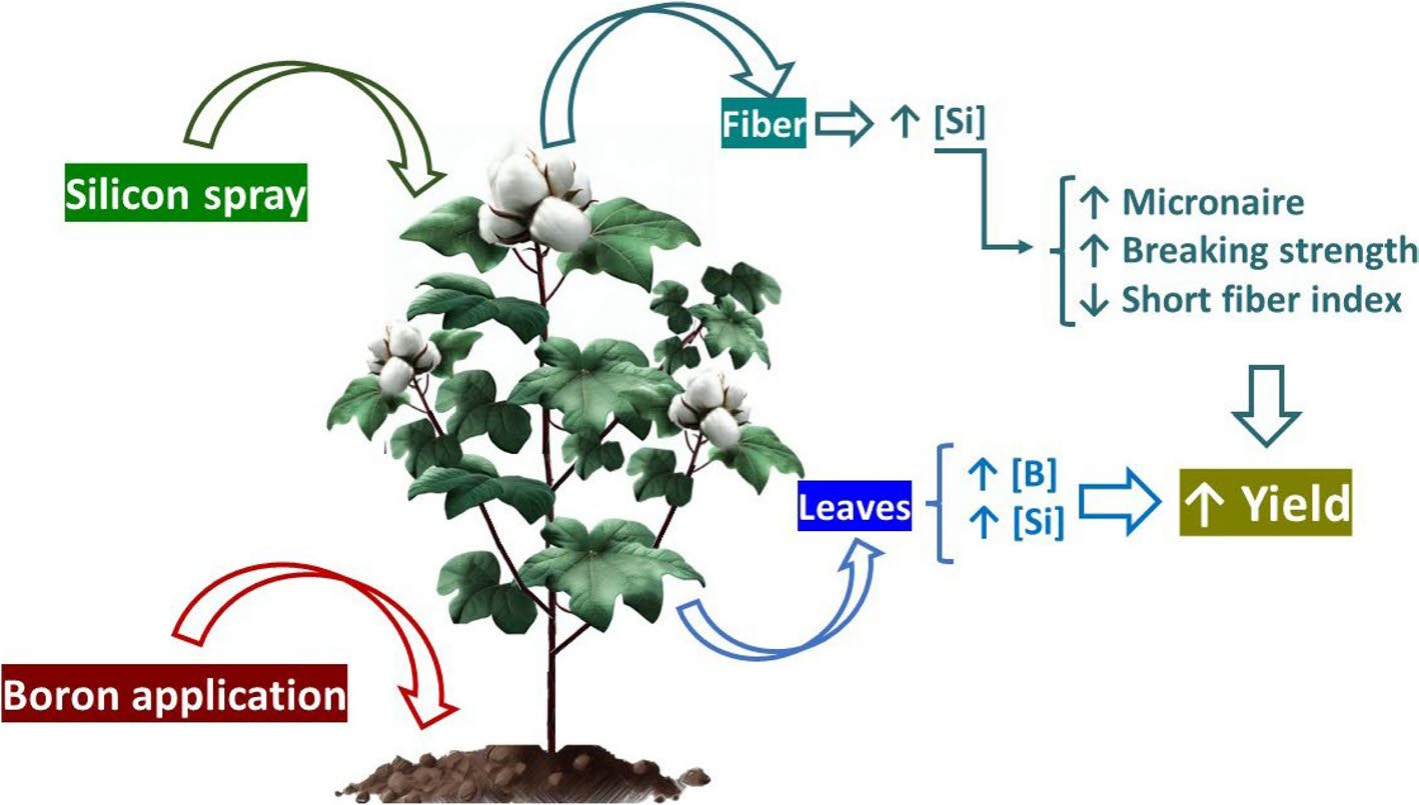
Diagram highlighting the main effects of the application of boron (B) via soil and silicon (Si) in the leaves. The results show an increase in the content of B in the leaf and Si in the leaves and in the fiber, leading to an increase in cotton yield, in cotton fiber micronaire and breaking strength and a reduction in the short fiber index

The results of this study support the hypothesis that soil-applied B increases cotton yield and fiber quality. In addition, the complementation with Si via foliar application increases fiber quality by enhance breaking strength and micronaire. In conclusion, the interaction between soil-applied B and foliar-applied Si is beneficial for cotton cultivation, resulting in high cotton yield with better fiber quality (Fig. 5).

This innovative and promising approach represents a crucial strategy to promote sustainability of tropical agriculture in B-deficient soils. The pioneering results demonstrated in this scientific note highlight the importance of prioritizing studies on different cultivars and specifically investigating deposition and biosilicification processes involving Si and B in cell wall components, mainly in cotton fibers, for a better understanding of this synergy.

## Data Availability

Data can be made available upon request to the corresponding author.

## References

[1] Li W, Shi Y, Zhu D, Wang W, Liu H, Li J (2021). Fine root biomass and morphology in a temperate forest are influenced more by the nitrogen treatment approach than the rate. Ecol Indic.

[2] Yang Y, Dou Y, Wang B, Xue Z, Wang Y, An S, et al. Deciphering factors driving soil microbial life‐history strategies in restored grasslands. iMeta. 2023;2(1):e66. 10.1002/imt2.66 PMC10989924 38868332

[3] Ahmed N, Ali MA, Danish  S, Chaudhry UK, Hussain S, Hassan W (2020). Role of macronutrients in cotton production. Cotton Production and Uses.

[4] Mehran M, Ashraf M, Shazad SM, Shakir MS, Azhar MT, Ahmad F (2023). Growth, yield and fiber quality characteristics of Bt and non-Bt cotton cultivars in response to boron nutrition. J Cotton Res.

[5] Ahmed N, Ali MA, Hussain S, Hassan W, Ahmad F, Danish S (2020). Cotton Production and Uses: Agronomy, Crop Protection, and Postharvest Technologies.

[6] Atique-ur-Rehman, Qamar R, Hussain A, Sardar H, Sarwar N, Javeed HMR (2020). Soil applied boron (B) improves growth, yield and fiber quality traits of cotton grown on calcareous saline soil. PLoS One.

[7] Souza Júnior JP, de Mello PR, Campos CNS, Oliveira DF, Cazetta JO, Detoni JA (2022). Silicon foliar spraying in the reproductive stage of cotton plays an equivalent role to boron in increasing yield, and combined boron-silicon application, without polymerization, increases fiber quality. Ind Crops Prod.

[8] dos Cordeiro LFS, dos Cordeiro CFS, Ferrari S (2022). Cotton yield and boron dynamics affected by cover crops and boron fertilization in a tropical sandy soil. Field Crops Res.

[9] Kumar S, Kumar D, Sekhon KS, Singh P, Phogat MV, Choudhary OP (2021). Soil application of boron impacts its solubility, yield and fibre quality of cotton in calcareous soils of North-western India. J Environ Biol.

[10] Kumar S, Kumar D, Sekhon KS, Choudhary OP (2018). Influence of levels and methods of boron application on the yield and uptake of boron by cotton in a calcareous soil of Punjab. Commun Soil Sci Plant Anal.

[11] Ahmed N, Abid M, Rashid A, Ali MA, Ammanullah M (2013). Boron requirement of irrigated cotton in a typic Haplocambid for optimum productivity and seed composition. Commun Soil Sci Plant Anal.

[12] Souza DMG, Lobato E. Cerrado: correção do solo e adubação [Cerrado: Soil correction and fertilization]. Brasília: Embrapa Informação Tecnológica; 2004.

[13] Epstein E (1994). The anomaly of silicon in plant biology. Proc Natl Acad Sci U S A.

[14] Kohli SK, Kaur H, Khanna K, Handa N, Bhardwaj R, Rinklebe J (2023). Boron in plants: uptake, deficiency and biological potential. Plant Growth Regul.

[15] de Souza Júnior JP, de Prado RM, dos Sarah MMS, Felisberto G (2019). Silicon mitigates boron deficiency and toxicity in cotton cultivated in nutrient solution. J Plant Nutr Soil Sci.

[16] Souza Júnior JP, de Prado MR, Campos CNS, Sousa Junior GS, Oliveira KR, Cazetta JO (2022). Addition of silicon to boron foliar spray in cotton plants modulates the antioxidative system attenuating boron deficiency and toxicity. BMC Plant Biol.

[17] de Souza Junior JP, de Prado RM, Silva Campos CN, da Sousa Junior GS, Costa MG, de Pádua Teixeira S (2023). Silicon modulate the non-enzymatic antioxidant defence system and oxidative stress in a similar way as boron in boron-deficient cotton flowers. Plant Physiol Biochem.

[18] Souza Júnior JP, de Mello PR, Ferreira Diniz J, de Farias Guedes VH, da Silva JLF, Roque CG (2022). Foliar application of innovative sources of silicon in soybean, cotton, and maize. J Soil Sci Plant Nutr.

[19] Barros TC, de Mello PR, Roque CG, Arf MV, Vilela RG (2019). Silicon and salicylic acid in the physiology and yield of cotton. J Plant Nutr.

[20] Prado R de M. Mineral nutrition of tropical plants. Switzerland: Springer Nature Switzerland AG; 2021.

[21] Cheng M, Cui Y, Yan X, Zhang R, Wang J, Wang X (2022). Effect of dual-modified cassava starches on intelligent packaging films containing red cabbage extracts. Food Hydrocoll.

[22] Yang Y, Liu L, Zhang P, Wu F, Wang Y, Xu C (2023). Large-scale ecosystem carbon stocks and their driving factors across Loess Plateau. Carbon Neutrality.

[23] Waghmare SV, Singh M (2017). Agrometeorological indices and correlation coefficient of Bt cotton under different growing environment. Int J Curr Microbiol Appl Sci.

[24] Santos HG, Jacomine PKT, Anjos LHC, Oliveira VA, Lumbreras JF, Coelho MR, et al. Sistema brasileiro de classificação de solos [Brazilian system of soil classification.]. 5 ed. Brasília: Embrapa Solos; 2018.

[25] Raij B Van, Andrade JC, Cantarella H, Quaggio JA. Análise química para availação da fertilidade de solos tropicais [Chemical analysis to assess the fertility of tropical soils]. Campinas: Instituto Agronômico de Campinas; 2001.

[26] Donagema GK, Campos DBVB, Calderano SB, Teixeira WG, Viana JH. Manual de métodos de análise de solo [Manual of soil analysis methods]. 2nd ed. Rio de Janeiro: Embrapa CNPTIA; 2011.

[27] Bataglia OC, Teixeira JPF, Furlani PR, Furlani AMC, Gallo JR. Métodos de análise química de plantas [Methods of chemical analysis of plants]. Campinas: Instituto Agronômico de Campinas; 1983.

[28] Kondörfer GH, Pereira HS, Nola A. Análise de silício: solo, planta e fertilizante [Silicon analyse: soil, plant and fertilizers]. Uberlândia: UFU; 2004.

[29] Fonseca RG, Santana JCF. Resultados de ensaio HVI e suas interpretações (ASTM D-4605) [HVI test results and their interpretations (ASTM D-4605)]. Circular T. Campina Grande: Embrapa Algodão; 2002.

[30] Barbosa JC, Maldonado Júnior W. AgroEstat: Sistema para análises estatísticas de ensaios agronômicos [AgroEstat: System for statistical and analysis of agronomic trials ]. 2015.

[31] Souza Júnior JP, de Mello Prado R, Soares MB, da Silva JLF, de Farias Guedes VH, dos Santos Sarah MM, et al. Effect of different foliar silicon sources on cotton plants. J Soil Sci Plant Nutr. 2020;21:1–9.

[32] Rabeh HA, El-Motaium RA, Badawy SH. Nano-silicon and boron foliar applications for promoting growth, yield, and fiber quality of Egyptian cotton ( *Gossypium barbadense* L.). J Plant Nutr. 2023;1:1–16.

[33] Bat-Erdene O, Szegő A, Gyöngyik M, Mirmazloum I, Papp I. Effects of silicon in plants with particular reference to horticultural crops - Review article. Int J Hortic Sci. 2021;27:95–105.

[34] Guerriero G, Hausman J-F, Legay S. Silicon and the plant extracellular matrix. Front Plant Sci. 2016;7:463. 10.3389/fpls.2016.00463 PMC4828433 27148294

[35] Bogiani JC, Rosolem CA (2012). Compared boron uptake and translocation in cotton cultivars. Rev Bras Cienc Solo.

[36] Fichhof WH, Silva RA, Oliveira LS, Silva RM. Management of biostimulant and silicon in mineral nutrition and quality of cotton fiber. J Agric Sci. 2018;10:476–84.

[37] Wainwright IM, Palmer RL, Dugger WM (1980). Pyrimidine pathway in boron-deficient cotton fiber. Plant Physiol.

[38] Boylston EK, Hebert JJ, Hensarling TP, Bradow JM, Thibodeaux DP (1990). Role of silicon in developing cotton fibers. J Plant Nutr.

